# Annotations

**Published:** 1902-03-01

**Authors:** 


### Annotations.

### Payment for Revaccination.

The proposal made by Mr. Scovell at the last meeting of the Metropolitan Asylums Board, that the public authorities should give some compensation to every person who suffered from want of work in consequence of revaccination, will certainly startle many people. We are so accustomed to see individuals put to all sorts of inconvenience for the public good without a penny-worth of compensation, or even a word of thanks being offered to them, that we have got into the way perhaps of regarding it as more incontrovertibly one's duty thus to suffer for one's neighbour without reward than is quite the case. When a man's child is taken from him "just because it had got a touch of scarlatina" and is mixed up for ten weeks or so with all sorts of other children, from whom it runs the risk of catching all sorts of other diseases, as happens constantly; when his house is for the same reason turned inside out and "disinfected," and when, if he or his elder children are engaged in certain industries, they are on the same plea prevented for a time from earning their wages—all this is done not for his own good, but for that of the community. Yet not only do we give him neither thanks nor compensation, but we actually regard him as an anti-social person if he rebels against his unhappy fate. Such is the law, and with such a public sentiment as the law expresses in favour of the individual being made to suffer for the crowd, it is inevitable that the suggestion that a workman ought to be compensated for the results of revaccination should be regarded by some as absolutely immoral, a sort of paying of blackmail to the anti-vaccinists. Still if we had a long pocket and a feeble law, and if we very much wanted vaccination and revaccination to be widely and efficiently performed, as we suppose is the wish of most sanitary authorities, it might be wiser to tackle the problem by aid of buns and shillings than by policemen and prosecutions. It is pandering to the enemy and compounding with evil no doubt, still it is a practical way out of a practical difficulty. The loss of wage-earning power which results from revaccination may not be great if we take the average, but now and again it falls heavily on the individual, and unfortunately we cannot say with confidence to any one individual "You will not suffer." But we can say "We will pay if you do," and we are inclined to agree with Mr. Scovell when he asserts that until that is done we shall not get the masses of the labouring population revaccinated.

### Dispensaries and Hospitals.

The treasurer of the Farringdon General Dispensary, at the annual meeting held a few days ago, stated that the decision of the Council of King Edward's Hospital Fund was to exclude all dispensaries from participation in the benefits of that fund. This is an entire misapprehension, seeing that King Edward's Hospital Fund was founded for the benefit of the hospitals, and that its object was to raise £100,000 a year for the benefit of the metropolitan hospitals from new sources, and by preference in small sums from a large number of new subscribers. To attempt this required much courage, and it is matter for satisfaction that the work has steadily progressed, is progressing, and promises to become entirely successful within a reasonable time. Should the £100,000 which the hospitals require be secured, but not till then, the question of deciding if any and what steps shall be taken to aid the dispensaries may arise. We fully recognise that the best-managed dispensaries are doing excellent work by visiting the sick poor in their own homes, but that work will not be helped, but rather hindered, and the sympathy which properly attaches to these institutions may be lessened, if the authorities do not avoid the mistake made by the treasurer of the Farringdon General Dispensary which might interfere with the attempt to place the hospitals upon a sound financial basis. Taken as a whole the metropolitan dispensaries are in a reasonably sound financial condition; the Farringdon General Dispensary, for example, with an expenditure of £561, had an income in 1901 of £557, and is not therefore in great need of funds. If the metropolitan dispensaries have a little patience we have no doubt that such assistance as they may need to enable them to do all the work entrusted to them will be forthcoming. The dispensaries must, however, avoid the error of interfering with or criticising the good work which is being done for the hospitals in the first instance. King Edward's Hospital Fund has rendered immense service to the voluntary system of medical relief in this country, and all who are labouring in any branch of that field of charity should gratefully recognise the fact and do their utmost to extend the revenue which is flowing into its coffers in increasing volume year by year.

